# Comparison of conventional ICSI and rescue ICSI in patients without severe male factor and poor oocyte yield

**DOI:** 10.3389/fendo.2025.1637404

**Published:** 2025-09-17

**Authors:** Mingzhao Li, Wennan Chen, Xia Xue

**Affiliations:** The ART Center, Northwest Women’s and Children’s Hospital, Xi’an, China

**Keywords:** fertilization failure, rescue ICSI, ICSI, c-IVF, reproductive outcomes

## Abstract

**Objective:**

The aim of this study was to explore the effectivity and safety of early rescue intracytoplasmic sperm injection (R-ICSI) in patients with poor oocyte yield and non-severe male factor.

**Methods:**

This study was a retrospective cohort analysis which included 604 conventional ICSI cycles and 116 early R-ICSI cycles at the Northwest Women’s and Children’s Hospital from February 2014 to December 2023. All patients were during their first assisted reproductive technologies (ART) cycle with 3–5 retrieved oocytes. The male partner had normal or mildly impaired sperm parameters. We compared the reproductive outcomes of conventional ICSI and early R-ICSI cycles in such patients.

**Results:**

We observed that there were no significant differences in the MII (86.75 versus 85.09%; *p* = 0.329) and two pronuclei (2PN) (71.82 versus 72.02%; *p* = 0.934) rates between conventional intracytoplasmic sperm injection (ICSI) and early R-ICSI groups. Following conventional ICSI, a total multi-pronuclei (MPN) rate of 1.02% was achieved, which was significantly lower than that of 6.33% after early R-ICSI (*p* < 0.001). There were no significant differences in the D3 good quality embryo (51.80 versus 49.67%; p = 0.499), D3 available embryo (82.28 versus 78.38%; *p* = 0.112) and blastocyst formation (65.15 versus 68.69%; *p* = 0.494) rates between the two groups. We also observed that there were no significant differences in the pregnancy (55.45 versus 50.50%; *p* = 0.357), clinical pregnancy (52.00 versus 46.53%; *p* = 0.312), ongoing pregnancy (44.91 versus 39.60%; p = 0.324) and live birth (42.73 versus 37.62%; *p* = 0.339) rates between the two groups.

**Conclusions:**

Despite the higher MPN rate, comparable outcomes can be achieved following early R-ICSI when compared to conventional ICSI for couples with non-severe male factor and poor oocyte yield.

## Introduction

Although the advancements in assisted reproductive technologies enable *in vitro* fertilization (IVF) to achieve fertilization rates ranging from 70% to 80%, unexpected total fertilization failure (TFF) still occurs in approximately 5% to 20% of conventional IVF (C-IVF) treatment cycles. When TFF occurs, no available embryos are obtained for transfer, leading to cycle cancellation. This situation is highly frustrating for patients and poses a significant challenge for clinicians. Although the causes of TFF remain incompletely understood, some studies have identified male factors, particularly sperm abnormalities, as significant contributors to this phenomenon (1, 2). However, other researches have demonstrated that sperm count, motility, and morphology are inadequate indicators of potential sperm-oocyte interaction (3, 4).

While the reliability of intracytoplasmic sperm injection (ICSI) has made it an appealing treatment option for infertile couples worldwide, including those without male factor infertility, its use in non-male factor cases remains controversial. It has been shown that ICSI significantly reduces the risk of TFF compared to C-IVF, while also decreasing cycle cancellation rates due to fertilization failure (5). Nevertheless, the arbitrary selection of sperm for injection may introduce additional risks and potential adverse outcomes (6). C-IVF preserves the natural sperm selection process during fertilization while avoiding potential mechanical oocyte damage associated with ICSI procedures. Nevertheless, multiple studies have demonstrated that patients with fewer than five oocytes are at a higher risk for TFF following C-IVF (7–9).

Multiple studies have demonstrated that ICSI had no obvious advantage in patients with normal semen parameters (10, 11). Meanwhiles, a recent study also indicated ICSI could not improve reproductive outcome compared with C-IVF in patients with non-severe male factor (12). The presented data corroborated that C-IVF should be recommended as the initial treatment option for patients with normal or near-normal semen parameters. C-IVF remains the recommended approach for most patients with a low oocyte yield and non-severe male factor infertility. Nevertheless, such patients may be at an increased risk of TFF following C-IVF.

It is crucial to balance the time-related risks associated with oocyte aging and multi-pronuclei (MPN) fertilization as both of them can lead to poor embryo quality. It’s still more challenging to perform early R-ICSI for couples who has normal semen analysis and poor oocyte yield. To salvage fertilization failure and mitigate the effects of oocyte aging, early rescue ICSI (R-ICSI) has been implemented 5–6 h post-insemination which demonstrate a promising clinical result (13). In this study, we aimed to explore the effectivity and safety of early R-ICSI in such cases.

## Materials and methods

### Study participants

**Figure 1:**
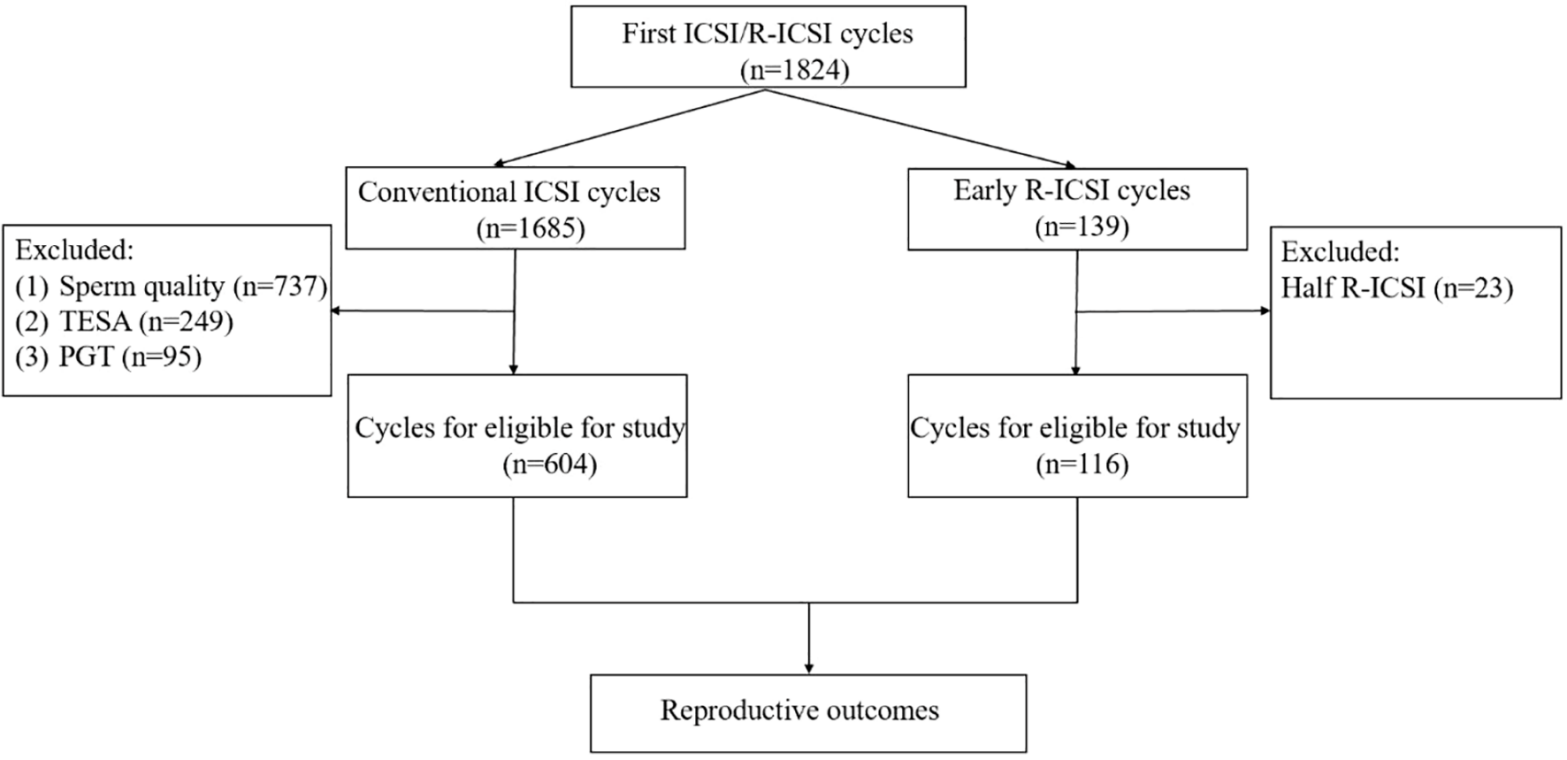
Eligibility assessment with exclusion criteria.

This study was a retrospective cohort analysis which included 604 conventional ICSI cycles and 116 early R-ICSI cycles at the Northwest Women’s and Children’s Hospital from February 2014 to December 2023. All patients were during their first assisted reproductive technologies (ART) cycle with 3–5 retrieved oocytes. The male partner had normal or mildly impaired sperm parameters with the processed semen sample (following density gradient purification) yielding at least 2 million progressively motile spermatozoa on the day of oocyte retrieval. TFF was defined as the absence of a second polar body in all mature oocytes. Near TFF was defined as fewer than 1/3 of mature oocytes exhibited a second polar body (second polar body rate < 33.33%). The cases of near TFF were excluded in this study. In this study, the conventional ICSI group was regarded as the control group, while early R-ICSI was considered the experimental group. The specific exclusion criteria were shown in **Figure 1**. All patients gave written informed consent and this study was approved by the Ethics Committee of Northwest Women’s and Children’s Hospital (No. 2023003).

### Ovarian stimulation protocol

All participants in our study underwent controlled ovarian hyperstimulation. The ovarian stimulation protocols in our reproductive medicine center include the GnRH agonist long protocol, GnRH agonist short protocol, and GnRH antagonist protocol, as detailed in previous literature (13). Notably, recombinant follicle-stimulating hormone (FSH) or urinary FSH and/or human menopausal gonadotropins were used with daily doses between 100 and 450 IU based on patients’ characteristics as calculated previously (13).

### C-IVF and early R-ICSI

C-IVF was performed 2-2.5 h after oocyte retrieval and each oocyte was incubated with approximately 40–000 sperm. Short-term insemination was adopted and the cumulus granule cells were peeled off 4.5–5 h post-fertilization. Oocytes were analyzed for the release of the second Pb at 5–6 h after the initial insemination. If there was no second Pb (TFF), R-ICSI was performed immediately on the oocytes with only one Pb observed. Our skilled ICSI operators injected the oocytes with only one Pb by the direct penetration technique.

### Fertilization check and embryo grading

Normal fertilization was confirmed 18–19 hours post-insemination through identification of two pronuclei (2PN) accompanied by second polar body extrusion. After 64–68 h of culture, the morphologic score was given for cleavage-stage embryos. The morphologic score of blastocysts were given on the fifth morning after oocyte retrieval. The detailed scoring criteria were based on our published literature (13). The D3 good quality embryos were graded I and II. The D3 available embryos were graded I, II, and III.

### Luteal support and embryo transfer

Three methods of luteal support are implemented in our center. I. Vaginal progesterone gel (90 mg q.d; Crinone, Serono, Hertfordshire, UK); II. Vaginal progesterone soft capsules (0.2 g t.i.d; Utrogestan, Besins, France); III. Intramuscular progesterone (60 mg q.d; Xianju, Zhejiang, China). Patients from both groups could select one of these three luteal support methods and receive oral progesterone (10 mg t.i.d; Dydrogesterone, Abbott Biologicals B.V., Amsterdam, Netherlands) simultaneously. The luteal support was maintained until week 10 of gestation.

The mucus in the cervical os was cleaned in advance with a cotton swab soaked in warm and humid saline. Embryos were transferred under ultrasound guidance with a transfer catheter (Limerick, Ireland). Pregnancy was defined as β-HCG value more than 50 mIU/ml after 12 days of transfer. Clinical pregnancy was characterized as the presence of an intrauterine gestational sac on ultrasonography during the first trimester. Ongoing pregnancy was defined as a clinical pregnancy that continued for a minimum of 12 weeks. Live birth was defined as the delivery of a live-born infant (> 24 weeks of gestation).

### Statistical analysis

Statistical analysis between groups in the case of continuous variables was performed with Student’s t test for data with normal distribution. Non-parametric Mann-Whitney U-test was performed for data with skewed distribution. Statistical analysis between groups in the case of categorical variables was expressed as number and percentage and Chi-square test or Fisher exact test was performed. The statistical analysis was performed with SPSS version 23 (IBM Corp.; NY, USA). A *p*-value of less than 0.05 was considered to indicate statistical significance.

## Results

### General characteristics of the enrolled patients

A total of 720 cycles were analyzed in this study, comprising 604 conventional ICSI cycles and 116 early R-ICSI cycles. Our data showed no significant differences in the aspects of female age, male age, BMI, basal FSH, Basal E_2_, infertile time, total Gn dosage, stimulation duration, the number of oocytes retrieved and the mean number of embryos transferred between conventional ICSI and early R-ICSI groups (*p*> 0.05) (**Table 1**).

**Table 1:** General characteristics of the enrolled patients.

Parameter	Conventional ICSI	Early R-ICSI	*P*-Value
Cycles (oocyte retrievals)	604	116	/
Female age (y)	32.34 ± 4.63	31.91 ± 4.30	0.389
Male age (y)	34.45 ± 5.75	33.81 ± 5.55	0.307
BMI for women (kg/m²)	23.12 ± 3.25	23.33 ± 4.79	0.685
Basal FSH (IU/L)	7.81 ± 2.61	7.19 ± 2.07	0.088
Basal E_2_ (pg/mL)	41.15 ± 20.52	41.25 ± 20.01	0.972
Infertile time (y)	4.17 ± 3.14	4.54 ± 3.52	0.291
Total Gn dosage (IU)	2643.39 ± 983.20	2650.81 ± 876.34	0.948
Stimulation duration (days)	10.23 ± 2.49	10.13 ± 2.46	0.561
Number of oocytes retrieved (n)	4.14 ± 0.80	4.20 ± 0.80	0.499
Mean number of embryos transferred (n)	1.45 ± 0.51	1.43 ± 0.50	0.780

### Embryo development in conventional ICSI and early R-ICSI groups

We observed that there were no significant differences in the MII (86.75 versus 85.09%; *p* = 0.329) and 2PN (71.82 versus 72.02%; *p* = 0.934) rates between conventional ICSI and early R-ICSI groups. Following conventional R-ICSI, a total ≥ 3PN rate of 1.02% was achieved, which was significantly lower than that of 6.33% after early R-ICSI (*p* < 0.001). There were no significant differences in the D3 good quality embryo (51.80 versus 49.67%; *p* = 0.499), D3 available embryo (82.28 versus 78.38%; *p* = 0.112) and blastocyst formation (65.15 versus 68.69%; *p* = 0.494) rates between the two groups (**Table 2**).

**Table 2:** Comparison of embryo development in conventional ICSI and early R-ICSI groups.

Parameter	Conventional ICSI	Early R-ICSI	*P*-Value
Cycles (oocyte retrievals)	604	116	/
MII rate (%, n)	86.75 (2161/2491)	85.09 (411/483)	0.329
2PN rate (%, n)	71.82 (1552/2161)	72.02 (296/411)	0.934
MPN rate (%, n)	1.02 (22/2161)	6.33 (26/411)	< 0.001
D3 good quality embryo rate (D3 good quality embryos/2PN)	51.80 (804/1552)	49.67 (147/296)	0.499
D3 available embryo rate (D3 embryos/2PN)	82.28 (1277/1552)	78.38 (232/296)	0.112
Embryos of extended culture to blastocyst-stage (n)	571	99	/
Blastocyst formation rate (%, n)	65.15 (372/571)	68.69 (68/99)	0.494

### Clinical outcomes in conventional ICSI and early R-ICSI groups

We further compared the clinical outcomes between conventional ICSI and early R-ICSI groups. Following early R-ICSI, the rate of cancelled transfers with no embryos available was 8.94%, which showed no significant difference with that of 12.93% after early R-ICSI (*p*> 0.05). We also observed that there were no significant differences in the pregnancy (55.45 versus 50.50%; *p* = 0.357), clinical pregnancy (52.00 versus 46.53%; *p* = 0.312), ongoing pregnancy (44.91 versus 39.60%; *p* = 0.324) and live birth (42.73 versus 37.62%; *p* = 0.339) rates between the two groups (**Table 3**).

**Table 3:** Comparison of clinical outcomes in conventional ICSI and early R-ICSI groups.

Parameter	Conventional ICSI	Early R-ICSI	*P*-Value
Cycles (Embryo transfers)	550	101	/
Cancelled transfers with no embryos available (%, n)	8.94 (54/604)	12.93 (15/116)	0.181
No. of transferred embryos (%, n)			0.829
n=1	55.27 (304/550)	56.44 (57/101)	
n=2	44.73 (246/550)	43.56 (44/101)	
Stage of transferred embryos (%, n)			0.511
Cleavage-stage	80.36 (442/550)	83.17 (84/101)	
Blastocyst-stage	19.64 (108/550)	16.83 (17/101)	
Pregnancy rate (%, n)	55.45 (305/550)	50.50 (51/101)	0.357
Clinical pregnancy rate (%, n)	52.00 (286/550)	46.53 (47/101)	0.312
Ongoing pregnancy rate (%, n)	44.91 (247/550)	39.60 (40/101)	0.324
Live birth rate (%, n)	42.73 (235/550)	37.62 (38/101)	0.339

### Embryo development and clinical outcomes according to the oocyte retrieval rate in conventional ICSI and early R-ICSI groups

For patients with ≥ 100% oocyte retrieval rate (ORR), the MII rate was significantly lower in ICSI group compared with that of early R-ICSI group (86.62 versus 92.66%; *p* = 0.025). There were no significant differences in the 2PN (73.71 versus 70.12%; *p* = 0.339), D3 good quality embryo (56.62 versus 56.52%; *p* = 0.984) and D3 available embryo (86.32 versus 81.74%; *p* = 0.195) rates between the two groups. Our data also showed no significant differences in the pregnancy (53.49 versus 55.81%; *p* = 0.777), clinical pregnancy (50.00 versus 53.49%; *p* = 0.672), ongoing pregnancy (43.80 versus 46.51%; *p* = 0.740) and live birth (43.02 versus 41.86%; *p* = 0.887) rates between the two groups (**Table 4**).

**Table 4:** Comparison of embryo development and clinical outcomes according to the oocyte retrieval rate (ORR) in conventional ICSI and early R-ICSI groups.

Parameter	ORR ≥ 100%			ORR < 100%		
	ICSI	Early R-ICSI	*P*	ICSI	Early R-ICSI	*P*
Cycles (Embryo transfers)	258	43	/	292	58	/
Female age (y)	33.22 ± 4.73	31.53 ± 4.75	0.067	31.55 ± 4.41	32.19 ± 3.95	0.529
MII rate (%, n)	86.62 (932/1076)	92.66 (164/177)	0.025	87.34 (1049/1201)	77.33 (191/247)	< 0.001
2PN rate (%, n)	73.71 (687/932)	70.12 (115/164)	0.339	75.31 (790/1049)	84.82 (162/191)	0.004
D3 good quality embryo rate (%, n)	56.62 (389/687)	56.52 (65/115)	0.984	52.53 (415/790)	50.62 (82/162)	0.657
D3 available embryo rate (%, n)	86.32 (593/687)	81.74 (94/115)	0.195	86.58 (684/790)	83.33 (135/162)	0.277
No. of transferred embryos (%, n)			0.535			0.959
n=1	57.75 (149/258)	62.79 (27/43)		53.08 (155/292)	53.45 (31/58)	
n=2	42.25 (109/258)	37.21 (16/43)		46.92 (137/292)	46.55 (27/58)	
Stage of transferred embryos (%, n)			0.418			0.918
Cleavage-stage	78.29 (202/258)	83.72 (36/43)		82.19 (240/292)	82.76 (48/58)	
Blastocyst-stage	21.71 (56/258)	16.28 (7/43)		17.81 (52/292)	17.24 (10/58)	
Pregnancy rate (%, n)	53.49 (138/258)	55.81 (24/43)	0.777	57.19 (167/292)	46.55 (27/58)	0.137
Clinical pregnancy rate (%, n)	50.00 (129/258)	53.49 (23/43)	0.672	53.77 (157/292)	41.38 (24/58)	0.085
Ongoing pregnancy rate (%, n)	43.80 (113/258)	46.51 (20/43)	0.740	45.89 (134/292)	34.48 (20/58)	0.110
Live birth rate (%, n)	43.02 (111/258)	41.86 (18/43)	0.887	42.47 (124/292)	34.48 (20/58)	0.259

For patients with a low ORR, the MII rate was significantly higher in ICSI group compared with that of early R-ICSI group (87.34 versus 77.33%; *p* < 0.001). However, we observed that the 2PN rate was significantly lower in ICSI group than that of early R-ICSI group (75.31 versus 84.82%; *p* = 0.004). There were no significant differences in the D3 good quality embryo (52.53 versus 50.62%; *p* = 0.657) and D3 available embryo (86.58 versus 83.33%; *p* = 0.277) rates between the two groups. Our data also demonstrated there were no significant differences in the pregnancy (57.19 versus 46.55%; *p* = 0.137), clinical pregnancy (53.77 versus 41.38%; *p* = 0.085), ongoing pregnancy (45.89 versus 34.48%; *p* = 0.110) and live birth (42.47 versus 34.48%; *p* = 0.259) rates between the two groups (**Table 4**).

## Discussion

Our results have demonstrated that comparable outcomes can be achieved following early R-ICSI when compared to conventional ICSI for couples with non-severe male factor and poor oocyte yield. Thus, we suggest that C-IVF may be the first choice of assisted reproductive technique for such patients.

When oocyte yield is limited, selecting the optimal fertilization method becomes crucial to maximize pregnancy success rates. Fang et al. showed ICSI did not improve the good-quality embryo rate or clinical pregnancy rate compared to C-IVF using semen with normal parameters in women with poor ovarian reserve (14). Isikoglu et al. concluded that low egg number is not an indication to perform ICSI in the presence of normal semen parameters (15). Meanwhiles, a recent study indicated that ICSI could not avoid the incidence of total fertilization failure and it might hamper the cumulative pregnancy rate in in poor responders (16). In non-male factor ART cycles, ICSI was not associated with improved pregnancy outcomes in older women with a low number of oocytes retrieved (17). The above data suggested that C-IVF should be the first choice of in patients with infertility with normal semen parameters and poor oocyte yield.

Nevertheless, the reproductive outcomes of such patients with fertilization failure following C-IVF have not been reported. Recently, multiple studies indicated that patients with fewer than five oocytes had a higher risk for fertilization failure following C-IVF (7–9). Tian et al. showed that semen parameters contribute to limited value in predicting TFF in unselected patients and oocyte yield is an important predictor for TFF (7). De souza et al. indicated that a decreased number of collected oocytes was the most important parameter associated with IVF failure in nonmasculine infertility (8). These results suggested that the study population had a high risk of fertilization failure in this research. However, the reproductive outcomes of such patients with fertilization failure following C-IVF have not been reported in current studies.

In cases of TFF, ICSI is routinely employed as a late remediation of unfertilized oocytes. In this research, the male partner had normal or mildly impaired sperm parameters for conventional ICSI. The primary population consists of patients with borderline semen quality or significant semen parameter fluctuations. If such patients undergo C-IVF treatment, TFF may occur, leading to cycle cancellation due to the absence of available embryos. This outcome can significantly increase both psychological and financial burdens for the patients (18). If the patients undergo ICSI treatment, it may introduce unnecessary or excessive treatment. And the application of ICSI in clinical practice should be carefully regulated as the potential long-term effects of ICSI on offspring remain a subject of ongoing debate (19). Multiple studies have shown that split insemination (combining C-IVF and ICSI for sibling oocytes) can be an effective strategy to prevent TFF while maintaining optimal embryo development potential in patients with borderline semen quality (20, 21). Nevertheless, the study population has a relatively low oocyte yield (only 3–5 oocytes retrieved), which presents significant challenges for implementing this treatment strategy.

Notably, we observed that R-ICSI could obtain similar reproductive outcomes compared conventional ICSI for patients with poor oocyte yield and no severe male factor. Thus, fertilization failure should not be a concern as early R-ICSI could achieve comparable clinical outcomes. It should be emphasized that this study was strictly limited to patients who yielded 3–5 oocytes during retrieval. The primary rationale stem is our clinical protocol against performing early R-ICSI for patients yielding only 1–2 oocytes. For such patients, long-time insemination was performed in order to minimize the MPN incidence resulting from wrong evaluation for fertilization evaluation. Historically, R-ICSI was routinely implemented for unfertilized oocytes 16–18 hours post insemination. However, the clinical outcome is always unsatisfactory which has been confirmed to be associated with oocyte aging (4). To salvage fertilization failure and mitigate the effects of oocyte aging, early R-ICSI has been implemented 5–6 h post-insemination which demonstrate a promising clinical result (13). Consistent with previous reported approach, early R-ICSI was also performed at this time point in our center.

For some patients with poor oocyte yield, a contributing factor is suboptimal oocyte retrieval rate. Only a scant number of oocytes were obtained from numerous matured follicles on the day of oocyte retrieval. It was confirmed that follicular flushing significantly increases the number of cumulus-oocyte complexes retrieved compared to single aspiration (22). Thus, Repeated flushing was performed to increase the oocyte retrieval rate. High flushing pressure might cause early rupture of the follicular wall, which results in oocytes damage as well as the outcome of embryos growth (23). Oocyte quality is widely recognized as the key factor governing embryo developmental competence in women (24).

For further analysis, the patients were allocated into subgroups according to the oocyte retrieval rate. We observed similar reproductive outcomes in conventional ICSI and early R-ICSI groups for patients with ≥ 100% predicted yield. For patients with below-anticipated oocyte retrieval, the conventional ICSI approach showed an increase rate of exceeding 10% for both clinical pregnancy and ongoing pregnancy compared to early R-ICSI intervention. Although no statistically significant differences were observed, which might be attributable to the limited R-ICSI cases in this research. And we observed that the MII rate was significantly higher in conventional ICSI group compared with that of early R-ICSI group. The way a single oocyte was affected might indicate how the whole cohort was affected, even if the other oocytes did not show the same characteristics. Low MII rates might indicate compromised cytoplasmic and nuclear maturation across the entire oocyte cohort. Complete nuclear and cytoplasmic maturation of oocytes is essential for the activation of oocytes at fertilization and the development of embryos. The preincubation of oocytes was considered to complete the final nuclear and cytoplasmic maturation of the oocyte. Ho et al. showed that the preincubation of oocytes for at least 2.5 h is beneficial to both IVF and ICSI outcomes by increasing the nuclear maturity of oocytes (25). In our center, C-IVF insemination is routinely performed at least 4 hours earlier than conventional ICSI and the oocyte preincubation period is typically limited to 2–3 hours in our protocol. Theoretically, the insemination timing should be delayed for such patients with low MII rate which might make negative effects on the embryo development and clinical outcomes. Nevertheless, we observed no significant differences in the D3 good quality embryo and available embryo rates between the conventional ICSI and early R-ICSI groups. A recent study demonstrated that the proportion of immature oocytes does not impact the outcomes of mature sibling oocytes (26). Therefore, the observed decline in success rates may not be attributable to embryo quality.

There are certain weaknesses in the current study that should be underlined. First, the primary drawback is the retrospective design and limited sample size of R-ICSI cases. Second, there may be some potential bias and confounders that cannot be excluded. Lastly, the cumulative live birth rate may be a more significant indicator and it is hard to calculate in this study. Nevertheless, few researches have explored the embryo development and clinical outcomes of early R-ICSI for such cases. Thus, the findings of the current study offer valuable insights for both clinicians and patients.

## Conclusions

Despite the higher MPN rate, comparable outcomes can be achieved following early R-ICSI when compared to conventional ICSI for couples with non-severe male factor and poor oocyte yield. Given the limited data and methodological constraints, further data accumulation is needed to obtain more reliable conclusions.

## Data Availability

The raw data supporting the conclusions of this article will be made available by the authors, without undue reservation.
